# Pharmacological Characterization of a Betaine/GABA Transporter 1 (BGT1) Inhibitor Displaying an Unusual Biphasic Inhibition Profile and Anti-seizure Effects

**DOI:** 10.1007/s11064-020-03017-y

**Published:** 2020-04-04

**Authors:** Maria E. K. Lie, Stefanie Kickinger, Jonas Skovgaard-Petersen, Gerhard F. Ecker, Rasmus P. Clausen, Arne Schousboe, H. Steve White, Petrine Wellendorph

**Affiliations:** 1 Department of Drug Design and Pharmacology, University of Copenhagen, Copenhagen, Denmark; 2 Department of Pharmaceutical Chemistry, University of Vienna, Vienna, Austria; 3 Department of Pharmacy, University of Washington, Washington, USA

**Keywords:** GABA uptake, GAT1, Homology modelling, Epilepsy, Q299L

## Abstract

Focal epileptic seizures can in some patients be managed by inhibiting γ-aminobutyric acid (GABA) uptake via the GABA transporter 1 (GAT1) using tiagabine (Gabitril®). Synergistic anti-seizure effects achieved by inhibition of both GAT1 and the betaine/GABA transporter (BGT1) by tiagabine and EF1502, compared to tiagabine alone, suggest BGT1 as a target in epilepsy. Yet, selective BGT1 inhibitors are needed for validation of this hypothesis. In that search, a series of BGT1 inhibitors typified by (1*R*,2*S*)-2-((4,4-bis(3-methylthiophen-2-yl)but-3-en-yl)(methyl)amino)cyclohexanecarboxylic acid (SBV2-114) was developed. A thorough pharmacological characterization of SBV2-114 using a cell-based [^3^H]GABA uptake assay at heterologously expressed BGT1, revealed an elusive biphasic inhibition profile with two IC_50_ values (4.7 and 556 μM). The biphasic profile was common for this structural class of compounds, including EF1502, and was confirmed in the MDCK II cell line endogenously expressing BGT1. The possibility of two binding sites for SBV2-114 at BGT1 was assessed by computational docking studies and examined by mutational studies. These investigations confirmed that the conserved residue Q299 in BGT1 is involved in, but not solely responsible for the biphasic inhibition profile of SBV2-114. Animal studies revealed anti-seizure effects of SBV2-114 in two mouse models, supporting a function of BGT1 in epilepsy. However, as SBV2-114 is apparent to be rather non-selective for BGT1, the translational relevance of this observation is unknown. Nevertheless, SBV2-114 constitutes a valuable tool compound to study the molecular mechanism of an emerging biphasic profile of BGT1-mediated GABA transport and the putative involvement of two binding sites for this class of compounds.

**Electronic supplementary material:**

The online version of this article (10.1007/s11064-020-03017-y) contains supplementary material, which is available to authorized users.

## Introduction

**Fig. 1:**
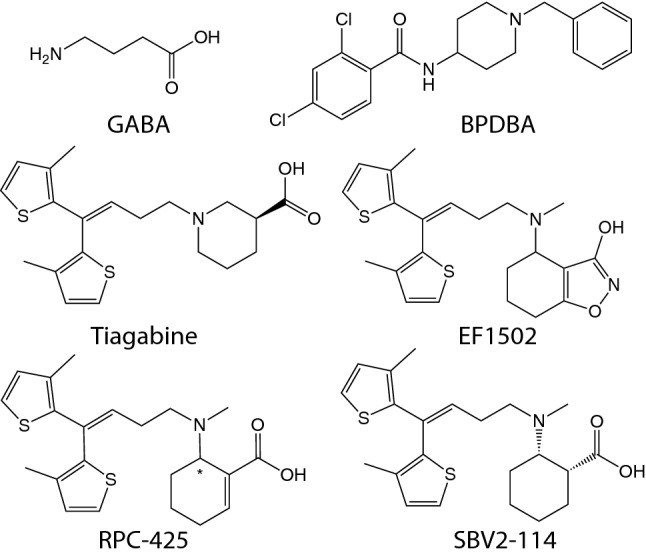
Structure of GABA, BPDBA (*N*-(1-benzyl-4-piperidinyl)-2,4-dichlorobenzamide), tiagabine, EF1502 ([N-[4,4-bis(3-methyl-2-thienyl)-3-butenyl]-3-hydroxy-4-(methylamino)-4,5,6,7-tetrahydrobenzo[d]isoxazol-3-ol]), RPC-425 (6-((4,4-bis(3-methylthiophen-2-yl)but-3-en-1-yl)(methyl)amino)cyclohex-1-ene-1-carboxylic acid), and SBV2-114 ((1*R*,2*S*)-2-((4,4-bis(3-methylthiophen-2-yl)but-3-en-yl)(methyl)amino)cyclohexanecarboxylic acid)

γ-Aminobutyric acid (GABA) is the main inhibitory neurotransmitter in the central nervous system (CNS) and is critical for maintaining normal neurotransmission. An imbalance between excitatory and inhibitory signalling is reported in several neurological disorders including epilepsy, Alzheimer’s disease, and ischemia [1–7]. Accordingly, enhanced GABAergic signalling may counterbalance the excessive excitatory neurotransmission and alleviate seizures in patients [8]. Fast inhibitory neurotransmission by GABA is mediated through ligand-gated ionotropic GABA_A_ receptors [9, 10], which are favoured therapeutic targets. Another way to enhance GABAergic signalling is by inhibition of the GABA transporters (GATs), thereby increasing extracellular GABA levels causing indirect inhibition [11, 12]. The GATs are transmembrane proteins comprised by four distinct transporters named GAT1, GAT2, GAT3 and BGT1 [13], according to the IUPHAR nomenclature [14]. GATs belong to the solute carrier 6 (SLC6) transporter family along with e.g. the serotonin transporter (SERT) for which crystal and cryo-electron microscopy structures have been solved [15, 16]. Due to the abundance of GAT1 in the CNS, most GAT research has been directed at this subtype, which led to the first antiepileptic drug, tiagabine (Gabitril®) (Fig. 1), targeting a GAT [17]. However, the substantial side effects of tiagabine [18, 19], together with studies of BGT1 inhibitors showing anti-seizure effects [20–23], have prompted research into this subtype as well. Specifically, the BGT1/GAT1 mixed inhibitor EF1502 and the BGT1 selective inhibitor RPC-425 (Fig. 1) [20] have been found to present anti-seizure effects in mouse models, suggesting a role for BGT1 in epilepsy [20–23]. Of particular relevance, BGT1 has been proposed to induce an extrasynaptic protecting mechanism following excitotoxic brain injury [24, 25]. Additionally, reported increases in BGT1 expression levels after epileptic insults in patients and animal models [8] support a potential role for BGT1 during certain pathological conditions. The role of BGT1 in seizure management, however, is questioned by the unchanged seizure threshold in BGT1 knock out (KO) mice [26], although compensatory mechanisms from the remaining GATs could have masked the effect of deleting BGT1 [27–29]. Thus, to shed light on the function of BGT1 in pathologies like epilepsy selective, brain-permeant compounds are desirable. To date, only a limited number of brain-permeant tool compounds, specifically targeting BGT1, are available [27]. In the further development of BGT1 ligands, the previous partial success of using β-amino acid-containing derivatives such as RPC-425 and EF1502, was extended. This led to the synthesis of (1*R*,2*S*)-2-((4,4-bis(3-methylthiophen-2-yl)but-3-en-yl)(methyl)amino)cyclohexanecarboxylic acid ((1*R*,2*S*)-5a, SBV2-114), derived from tiagabine (Fig. 1) [20, 30]. SBV2-114 was found to be an inhibitor of BGT1 with a potency in the mid μM range and with > 13 fold selectivity for BGT1 over the other GAT subtypes [30]. Looking further into the uptake inhibition profile revealed an unusual biphasic inhibition profile of SBV2-114 at BGT1. Also, anti-seizure effects of SBV2-114 in two mouse models was demonstrated, substantiating the involvement of BGT1 in epilepsy. To our knowledge this is the first example of a biphasic ligand profile at a GABA transporter and the class of compounds including SBV2-114, may ultimately contribute to our understanding of GAT transport mechanisms and further the pharmacological studies of BGT1 as a drug target in epilepsy.

## Materials and Methods

### Materials

Dulbecco’s Modified Eagle Medium (DMEM) with GlutaMAX-I, Hank’s Balanced Salt Solution (HBSS), fetal bovine serum (FBS), and penicillin–streptomycin (P/S) were purchased from Life Technologies (Paisley, UK). PolyFect Transfection Reagent was purchased from Qiagen (West Sussex, UK). HEPES (4-(2-hydroxyethyl)piperazine-1-ethanesulfonic acid), Poly-d-lysine (PDL), ATP (adenosine 5′-triphosphate**),** U-73122, CaCl_2_, and MgCl_2_ were purchased from Sigma-Aldrich (St. Louis, MO, USA). 1,2-Bis(2-aminophenoxy)ethane-*N*,*N*,*N*′,*N*′-tetraacetic acid tetrakis(acetoxymethyl ester) (BAPTA/AM) was purchased from Millipore (Billerica, MA, USA) while BAPTA, Fluo-4 loading dye, Pluronic F-127, and probenecid were purchased from ThermoFisher Scientific (Waltham, MA USA). 1-[3-(9*H*-Carbazol-9-yl)propyl]-4-(2-methoxyphenyl)-4-piperidinol (NNC 05–2090) and A 804598 were purchased from Tocris Bioscience (Bristol, UK). [2,3-^3^H(N)]GABA (35.0 Ci/mmol) and Micro-Scint-20 were purchased from PerkinElmer (Boston, MA, USA).

(*R*)-4-((4,4-bis(3-methylthiophen-2-yl)butyl)(methyl)amino)-4,5,6,7-tetrahydrobenzo[*d*]isoxazol-3-ol ((*R*)-EF1502), (1*R*,2*S*)-2-((4,4-bis(3-methylthiophen-2-yl)but-3-en-yl)(methyl)amino)cyclohexanecarboxylic acid ((SBV2-114), (1*R*,2*S*)-5a), (1*R*,2*R*)-2-((4,4-bis(3-methylthiophen-2-yl)but-3-en-1-yl)(methyl)amino)cyclohexane-1-carboxylic acid ((1*R*,2*R*)-4a), (1*S*,2*R*)-2-((4,4-bis(3-methylthiophen-2-yl)but-3-en-1-yl)(methyl)amino)cyclohexane-1-carboxylic acid ((1*S*,2*R*)-5a), (1*S*,2*R*)-2-((3-(10,11-dihydro-5*H*-dibenzo[*a*,*d*][7] annulen-5-ylidene)propyl)(methyl)amino)cyclohexane-1-carboxylic acid ((1*S*,2*R*)-5c), and *N*-(1-benzyl-4-piperidinyl)-2,4-dichlorobenzamide (BPDBA) were synthesized in-house as previously described [23, 30, 31]. The Madin-Darby *canine* kidney (MDCK) II cell line was kindly provided by Dr. Hanne Borger Rasmussen (University of Copenhagen, Denmark).

### Cell Culturing and Transient GAT Expression

The HEK-293 and Flp-In CHO cell lines stably expressing *mouse* (m) and *human* (h) GATs, respectively, have been described previously and were cultured accordingly [31–33]. The tsA201 cell line was used for transient expression while the MDCK II cell line express BGT1 endogenously [34]. Both cell lines were cultured as described [33, 35]. tsA201 cells were transfected with DNA constructs (8 μg per 10 cm dish if not otherwise mentioned) using 40 μL PolyFect transfection reagent according to the manufacturer’s protocol (Qiagen, Venlo, Netherlands). The DNA constructs encoding hBGT1, hGAT1-3 [36], the mutated constructs hBGT1 Q299 and hGAT3 L314Q described previously [37], and hBGT Y453A were synthesized and sequence validated by Genscript (NJ, USA).

### [^3^H]GABA Uptake Assays

The [^3^H]GABA competition uptake assay was performed as previously described [36]. A minor change, however, was introduced for uptake in MDCK II cells, in which 100 nM [^3^H]GABA was used for a 15 min incubation period at 37 °C instead of 30 nM [^3^H]GABA and 3 min incubation. Studies related to the inhibition curves with GABA and BPDBA at hBGT1 were obtained both with and without the presence of 10 μM ATP during the [^3^H]GABA uptake assay.

To examine the influence of Ca^2+^ on the functional activity, tsA201 cells were pre-incubated with 25 μM BAPTA, 25 μM BAPTA/AM, 5 μM U-73122 or 1 μM A804598 in assay buffer for 30 min prior to the [^3^H]GABA uptake to ensure that the compounds reached their site of action. All compounds, except BAPTA/AM, were also present at the same concentration during the course of the actual [^3^H]GABA uptake assay.

The [^3^H]GABA uptake data was normalized to the percentage of total uptake in the individual experiments in the presence of the lowest compound concentration or no compound present. Data presented is the pooled data of at least three independent experiments with three technical replicates. Concentration–response curves (CRCs) were fitted with GraphPad Prism (version 8.2.1, Yosemite, GraphPad Software, San Diego, CA, USA) by non-linear regression to the sigmoidal concentration response model:

$$Y=\text{Bottom}+\frac{(\text{Top}-\text{Bottom})}{{1+10}^{\left(\text{LogIC}_{50}-X\right)*nH}}$$

where Top is the Top plateau, Bottom is the Bottom plateau, X is the logarithm of the concentration of the compound, LogIC_50_ is the concentration giving a response halfway between Top and Bottom,and nH is the Hill slope.

And the biphasic model:

$$\text{Section}1=(\text{Top}-\text{Bottom}){*}\frac{\text{Frac}}{{1+10}^{\left(\text{LogIC}_{50\_1}-X\right){*}nH1}},$$

$$\text{Section}2=(\text{Top}-\text{Bottom})*\frac{(1-\text{Frac})}{{1+10}^{\left(\text{LogIC}_{50\_2}-X\right)*nH2}},$$

$$Y=\text{Bottom}+\text{Section}1+\text{Section}2$$

where Bottom and Top are the plateaus at the left and right ends of the curve, in the same units as Y. LogIC_50__1 and LogIC_50__2 are the concentrations that give half-maximal inhibitory effects. nH1 and nH2 are the Hill slopes and Frac is the proportion of maximal response due to the more potent phase.

The best fit for CRCs obtained with the [^3^H]GABA uptake assay was identified by the extra-sum-of-squares F test (for convenience referred to as F test).

### Modeling

A hBGT1 homology model based on the hSERT crystal structure PDB ID 5I73 [15] was used from Jørgensen et al. [37]. Protein and ligand preparation were performed with PrepWiz [38] and LigPrep [39], respectively, in the Schrödinger Suite 2015-2. Flexible side chain docking was performed with GOLD 5.2.2 [40] into the orthosteric and allosteric site corresponding to the orthosteric and allosteric sites in the crystal structure 5I73 (hBGT1 orthosteric site residues (flexible side chains in italics); *Glu52*, Ile53, Ile54, Gly55, Ile125, *Glu126*, Leu129, *Asn130*, Tyr132, *Tyr133*, *Phe293*, Ser294, Phe295, Ala296, *Gln299*, Cys301, Gly392, *Ser395*, Gln396, Val398, *Cys399*, *Ser456*, *Ser457*, Leu461; allosteric site residues: *Trp60*, Leu56, *Arg61*, *Tyr132*, *Tyr133*, Ile136, Asp286, *Thr289*, Gln290, *Phe293*, Ser294, *Phe448*, *Asp452*, *Tyr453*, *Ser457*, Gly458, Ile459, Ser516, Tyr520, Thr521). 100 SBV2-114 poses per site were generated and scored according to the internal GOLD 5.2.2 ChemPLP scoring function [37] as well as rescored with GBVI/WSA dG and London dG scoring functions implemented in Moe [41]. Visual inspection of all poses was performed with Moe [41].

### Fluo-4 Ca^2+^-Assay

SBV2-114 was tested for its ability to induce Ca^2+^ increases in tsA201 cells transiently expressing hBGT1 or hGAT1 by the Fluo4 Ca^2+^-assay as previously described with minor changes [42]. In brief, 4 μM Fluo-4 loading dye was mixed with 0.004% Pluronic F-127 and dissolved in HBSS buffer with 20 mM HEPES, 1 mM CaCl_2_ and 1 mM MgCl_2_ (HBSS-HEPES) supplemented with 2.5 mM probenecid. 50 μL of the loading buffer was added to each 96 well before incubating the cells 60 min at 37 °C. Cells were then washed with HBSS-HEPES buffer before changes in intracellular Ca^2+^ were recorded for 190 s. on a Flexstation 3 Benchtop multi-mode microplate reader (Molecular Devices, Sunnyvale, CA, USA) at 37 °C with an λ_ex_ of 485 nm and λ_em_ of 525 nm. Emission was recorded for 20 s. before adding the compounds (at 4 × concentrations) to the HBSS-HEPES buffer to determine the baseline. The changes in fluorescence in the Fluo-4 Ca^2+^-assay were normalized to the percentage of the maximum response elicited by 100 μM ATP in the individual experiments. Data presented is the pooled data of at least three independent experiments with three technical replicates. Changes in fluorescence units (ΔFU) were determined as the maximum fluorescent signal within the 190 s minus the average of the baseline.

### Animals

Male audiogenic-susceptible (AGS) Frings mice were obtained from an in-house breeding facility at the University of Utah (Salt Lake City, UT, USA). Male CF-1 mice for the maximal electroshock (MES) test and the subcutaneous pentylenetetrazol (s.c.PTZ) seizure threshold test were purchased from Charles River (Kingston, WA, USA). All mice were housed in a temperature, humidity, and light (on at 6:00 AM) controlled facility at the University of Utah with access to ad libitum food and water except during testing. All procedures were carried out at the University of Utah in concordance with the National Institutes of Health Guidelines for the Care and Use of Laboratory Animals and under approval from the University of Utah’s Institutional Animal Care and Use Committee. The mice were euthanized in accordance with Public Health Service policies on the humane care of laboratory animals.

### Audiogenic Seizures

The ability of SBV2-114 to prevent sound-induced seizures was studied in AGS Frings mice. The seizures were induced as previously described [20]. In brief, Frings mice were placed in a Plexiglas cylinder (diameter 15 cm, height 18 cm) connected to an audio transducer and exposed to a sound stimulus of 110 decibel (11 kHz) for a duration of 20 s. SBV2-114 (50 and 120 mg/kg, n = 4) was injected intraperitoneal (i.p.) at various time points to determine the time-to peak (TPE). The mice were considered protected if they failed to display full hind limb tonic extension seizures. All Frings mice were pre-screened the day prior to testing and only those mice that displayed a tonic extension seizure in response to the sound stimulus were included in the SBV2-114 studies.

### Maximal Electroshock (MES) Test

The ability of SBV2-114 to prevent electroshock-induced seizures was assessed using the MES test. This is a model of generalised tonic–clonic seizures, and was conducted as described previously [21]. In brief, a drop of anaesthetic/electrolyte solution (0.5% tetracaine hydrochloride in 0.9% saline) was applied to the cornea of each animal prior to placement of the silver-coated corneal electrodes. An alternating current of 50 mA (60 Hz) was delivered for 0.2 s to the cornea of the mouse using a Woodbury–Davenport stimulator. The supramaximal current applied to the cornea was sufficient to induce maximal tonic extension seizures in all non-treated mice. Separate groups of mice were treated with increasing doses of SBV2-114 (50–200 mg/kg, i.p., n = 3–7) and were exposed to the MES stimulus at the predetermined SBV2-114 TPE; i.e., 15 min. Those mice not displaying full hind limb tonic extension seizures were considered protected.

### Subcutaneous Pentylenetetrazol Seizure Test

SBV2-114 was tested for its ability to prevent a minimal clonic seizure in the s.c.PTZ seizure test, which is a test of generalized myoclonic seizures. The s.c.PTZ seizure test was carried out as previously described [21], and SBV2-114 was injected i.p. at a dose of 150 mg/kg (n = 8). At the TPE, mice were challenged with a s.c. dose of PTZ (85 mg/kg) and observed for 30 min for the presence or absence of a minimal clonic seizure characterized by minimal forelimb clonus and/or vibrissae twitching. Mice not displaying this phenotypic behaviour were considered protected.

### Behavioural Impairment

Minimal motor impairment of mice receiving SBV2-114 was evaluated using the rotarod procedure [43]. Impairment was noted if the mice failed to maintain their equilibrium for 1 min in three consecutive trials on a one-inch rotating rod (6 rpm). The rotarod test was performed just prior to the three seizure tests.

### Quantification of Anti-seizure Efficacy and Behavioural Impairment

Probit analysis [21] was used to analyse the efficacy and tolerability results obtained from the dose–response studies and to calculate the median effective (ED_50_) dose, the median toxic dose (TD_50_), and the 95% confidence interval.

### Screening for Selectivity

The selectivity of SBV2-114 for BGT1 for 42 different neuroreceptors and transporters was tested by examining the affinity of SBV2-114 in radioligand binding assays at the National Institute of Mental Health’s Psychoactive Drug Screening Program (NIMH PDSP, Contract # HHSN-271-2008-00025-C). SBV2-114 was tested at a concentration of 10 μM followed by full curves if inhibition exceeded 50% at 10 μM.

### Statistical Analysis

All statistical and pharmacological analyses were performed using GraphPad Prism (version 8.2.1, Yosemite, GraphPad Software, San Diego, CA, USA), and appropriate statistical tests are specified accordingly.  Level of significance was set at P < 0.05.

## Results

### Identification of a Biphasic Inhibition Profile of SBV2-114 at BGT1

**Fig. 2:**
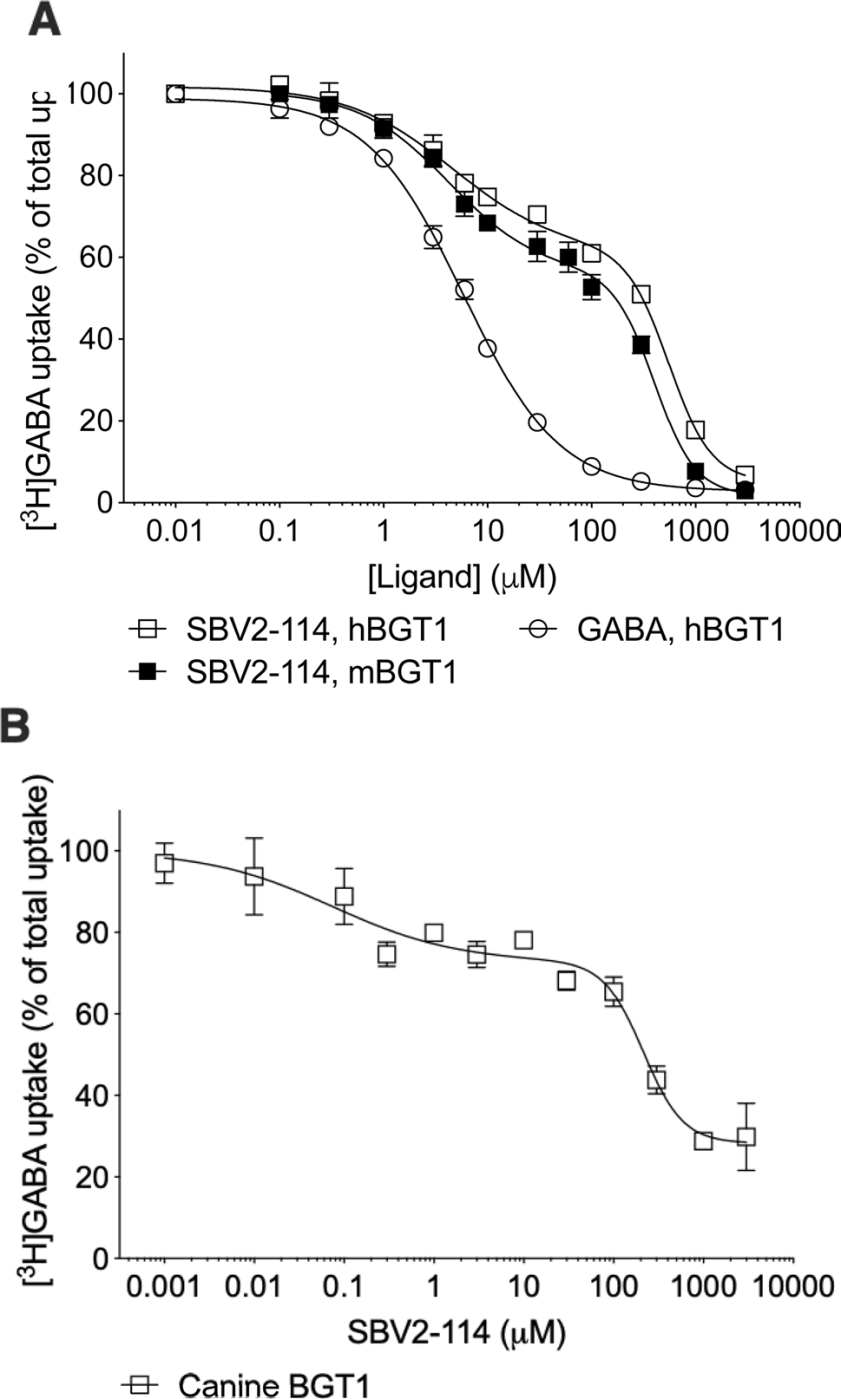
Inhibitory activity of SBV2-114 at *mouse, human* and *canine* BGT1. (**a**) SBV2-114 and GABA were tested for their ability to inhibit uptake of 30 nM [^3^H]GABA for 3 min at mBGT1 and hBGT1 in HEK-293 and tsa201 cells, respectively, and (**b**) 100 nM [^3^H]GABA for 15 min at *canine* BGT1 in MDCK II cells. The experiments were performed in triplicate in four-five independent experiments and depicted as normalized means ± S.E.M. The data fitting is based on the preferred model according to the extra-sum-of-squares F test (see Table 1 for details). Typical counts ranged from 100 to 8000 CPM at hBGT1 and mBGT1 and 500 to 2000 CPM at canine BGT1

Previous studies in our group have revealed that SBV2-114 has a potency in the mid μM range for mBGT1 and displays about 26, 43 and 13 times reduced inhibitory potency at mGAT1, mGAT2 and mGAT3, respectively [30]. In this study, a thorough pharmacological characterization of SBV2-114 using the competitive [^3^H]GABA uptake assay was performed. This entailed 12 concentrations to generate a detailed CRC. This study revealed that SBV2-114 inhibited mBGT1 (stably expressed in HEK-293 cells) and hBGT1 (transiently expressed in tsA201 cells) in an unusual biphasic manner (confirmed by F tests) with IC_50_ values in the low (m/hBGT1: 3.8/4.7 μM) and high μM range (m/hBGT1: 402.7/555.9 μM) corresponding to the high and low affinity components, respectively (Fig. 2a and Table 1). The % inhibition (Frac value) at the low IC_50_ was 45% at mBGT1 and 42% at hBGT1. This biphasic behaviour was not seen in previously performed 8-point mBGT1 inhibition CRCs [30]. In comparison, the endogenous ligand GABA, the BGT1 inhibitor BPDBA (Fig. 2a, Supplemental Fig. 2b), and the conventional GAT1 inhibitor tiagabine all inhibit [^3^H]GABA uptake in a clearly monophasic manner [36]. Furthermore, the IC_50_ values of SBV2-114 were in the same range regardless of the amount of plasmid DNA used for the transient expression of hBGT1 (Supplemental Table 1) indicating that the biphasic inhibition profile at BGT1 was not influenced by the expression level of BGT1.

**Table 1:** Inhibitory activity of SBV2-114 at *mouse*, *human* and *canine* BGT1. SBV2-114 was tested for its ability to inhibit uptake of [^3^H]GABA (see Fig. 1 for details)

[^3^H]GABA uptake at BGT1			Extra-sum-of-squares F test				
Species^a^	IC_50_ (pIC_50_ ± S.E.M.) (μM)		P value	F value	DFn, DFd	Frac	nH_1_, nH_2_
*Mouse*	3.8 (5.42 ± 0.08)	408.3 (3.39 ± 0.03)	< 0.0001	10.6	3, 53	0.45	− 0.62, − 2.61
*Human*	4.7 (5.28 ± 0.10)	555.9 (3.26 ± 0.03)	< 0.0001	11.2	3, 53	0.42	− 0.86, − 2.13
*Canine*	0.1 (6.98 ± 0.79)	250.6 (3.60 ± 0.10)	0.0067	5.7	2, 41	0.38	− 0.62, − 1.97

All experiments were performed in triplicate in four-five independent experiments. The data fitting is based on the preferred model according to the extra-sum-of-squares F test, and the associated P, F, DFn, DFd, Frac values, and nH_1_, nH_2_ are indicated

^a^*mouse* BGT1 stably expressed in HEK-293 cells, *human* BGT1 transiently expressed in tsA201 cells, and *canine* BGT1 endogenously expressed in MDCK II cells

To investigate if the biphasic inhibition profile of SBV2-114 was a consequence of recombinant overexpression in the heterologous expression system, SBV2-114 was tested at MDCK II cells endogenously expressing *canine* BGT1 [34]. In this study with MDCK II cells, SBV2-114 retained its biphasic inhibition profile (P = 0.0067, F test) at BGT1 with IC_50_ values of 0.1 μM and 250.6 μM (Fig. 2b and Table 1) supporting the finding that the biphasic inhibition profile of SBV2-114 at BGT1 is not a phenomenon only seen in overexpressed cells and is independent of the species.

To ascertain whether the biphasic CRC of SBV2-114 at BGT1 was caused by kinetic factors, e.g. by a slower on-kinetics, tsA201 cells expressing hBGT1 were pre-incubated for 10 min with SBV2-114 prior to the [^3^H]GABA uptake assay. This had no impact on the curve fitting and the biphasic fit was still preferred (P < 0.0001, F test, data not shown) although pre-incubation reduced the % inhibition at the low IC_50_ as the Frac value decreased from 0.45 to 0.34.

To test whether the biphasic inhibition profile was confined to BGT1, the detailed pharmacological characterization of SBV2-114 was extended to mGAT1-3 stably expressed in HEK-293 cells [32] and hGATs stably expressed in Flp-In CHO cells [36]. It is clear that SBV2-114 did not inhibit the m/hGAT1-3 in a typical monophasic manner (Supplemental Tables 2 and 3). Specifically, the fitting of the inhibition profile of SBV2-114 was predominantly ambiguous, meaning neither fitted to a monophasic or biphasic profile, at mGAT1-3-HEK-293 with IC_50_ values > 1000 μM (Supplemental Table 2). SBV2-114 also produced ambiguous fitting results at the hGATs (Supplemental Table 3). In detail, SBV2-114 inhibited the [^3^H]GABA uptake at hBGT1-CHO in a biphasic manner in two out of four experiments, and with a reduced % inhibition at the low IC_50_ (Frac = 0.10). Additionally, SBV2-114 displayed a biphasic inhibition profile at hGAT3, but also with a reduced Frac value compared to hBGT1-tsA, and in one out of four experiments at GAT2 (Supplemental Table 3). These data collectively show that SBV2-114 generally inhibits the [^3^H]GABA uptake by m/hGATs in an atypical manner.

### Several Structurally Related SBV2-114 Analogues Also Inhibit BGT1 in a Biphasic Manner

To investigate whether the biphasic inhibition profile at BGT1 is unique to SBV2-114, we also characterized the inhibition profile of a number of different compound classes in the [^3^H]GABA uptake assay (Table 2). We tested three compounds that all contain a β-amino acid moiety like SBV2-114, but with varying stereochemistry and different di-aromatic lipophilic side-chains including (1*R*,2*R*)-4a, (1*S*,2*R*)-5a, and (1*S*,2*R*)-5c [30]. (*R*)-EF1502 was also included in the detailed pharmacological characterization, with no prior notion of a biphasic inhibition profile, as it is structurally highly similar to SBV2-114. Finally, two BGT1 inhibitors belonging to other compound classes namely BPDBA [31] and NNC 05-2090 [44] were included (Fig. 1). Convincingly, all compounds containing a β-amino acid moiety with di-aromatic lipophilic side-chains including (*R*)-EF1502 showed a biphasic CRC at hBGT1 transiently expressed in tsA201 cells. The most potent component of the biphasic response ranged from 2.5–11.2 μM inhibiting a fraction of 0.19–0.42, where the largest inhibition was observed with SBV2-114. The high IC_50_ values ranged from 190.5 to 818.5 μM (Table 2). (*R*)-EF1502 and SBV2-114 were the compounds with the highest F value, respectively, and hence statistically, the ones with the most distinct biphasic CRCs. BGT1 inhibitors, not comprising a β-amino acid moiety attached to di-aromatic lipophilic side-chains, such as BPDBA and NNC 05–2090, did not inhibit hBGT1 in a biphasic manner (Table 2, Supplemental Fig. 2b).

**Table 2:**
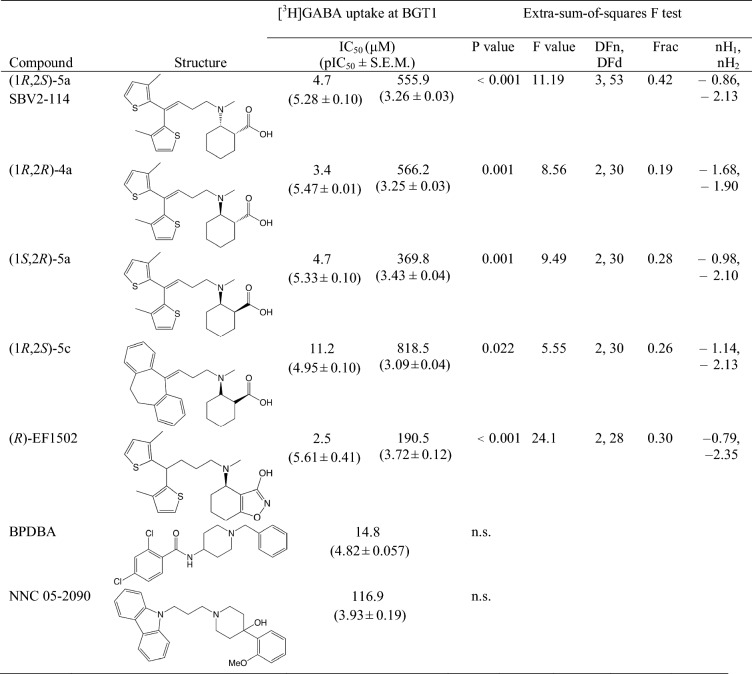
Chemical structures of SBV2-114, selected SBV2-114 analogues and other tested BGT1 inhibitors and their inhibitory activity at hBGT1 transiently expressed in tsA201 cells

The compounds were tested for their ability to inhibit uptake of 30 nM [3H]GABA for 3 min. All experiments were performed in triplicates in three–five independent experiments. The curve fittings are based on the preferred model according to the extra-sum-of-squares F test with the associated P, F, DFn, DFd, Frac values, and nH_1_, nH_2_ indicated accordingly. Non-significant (n.s.)

### Investigations into the Possible Mechanisms of Action

Having verified the biphasic response of SBV2-114 and structurally related compounds at BGT1 in the [^3^H]GABA uptake assay, we wanted to address the molecular details. To this end, the possibility of SBV2-114 binding to two non-equal binding sites in BGT1 was investigated. Further, the influence of Ca^2+^ on the inhibition profile was examined due to the link between intracellular Ca^2+^ changes and altered [^3^H]GABA uptake [45].

### Could SBV2-114 Bind to Two Non-equal Binding Sites?

The fact that SBV2-114 clearly inhibits BGT1 in a biphasic manner with two distinct IC_50_ values could suggest that SBV2-114 possibly binds to two different sites in BGT1. This is in line with recent crystallographic evidence that the antidepressant escitalopram not only binds to the orthosteric pocket of the *human* SERT, but also to an allosteric pocket in the extracellular vestibule above the orthosteric site [15]. Since hSERT is highly related to hBGT1 (44% sequence identity), both belong to the SLC6A family as well as share the same fold, it is very likely that a similar allosteric binding site exist in BGT1 and the other GATs [13, 15, 37, 46, 47].

**Fig. 3:**
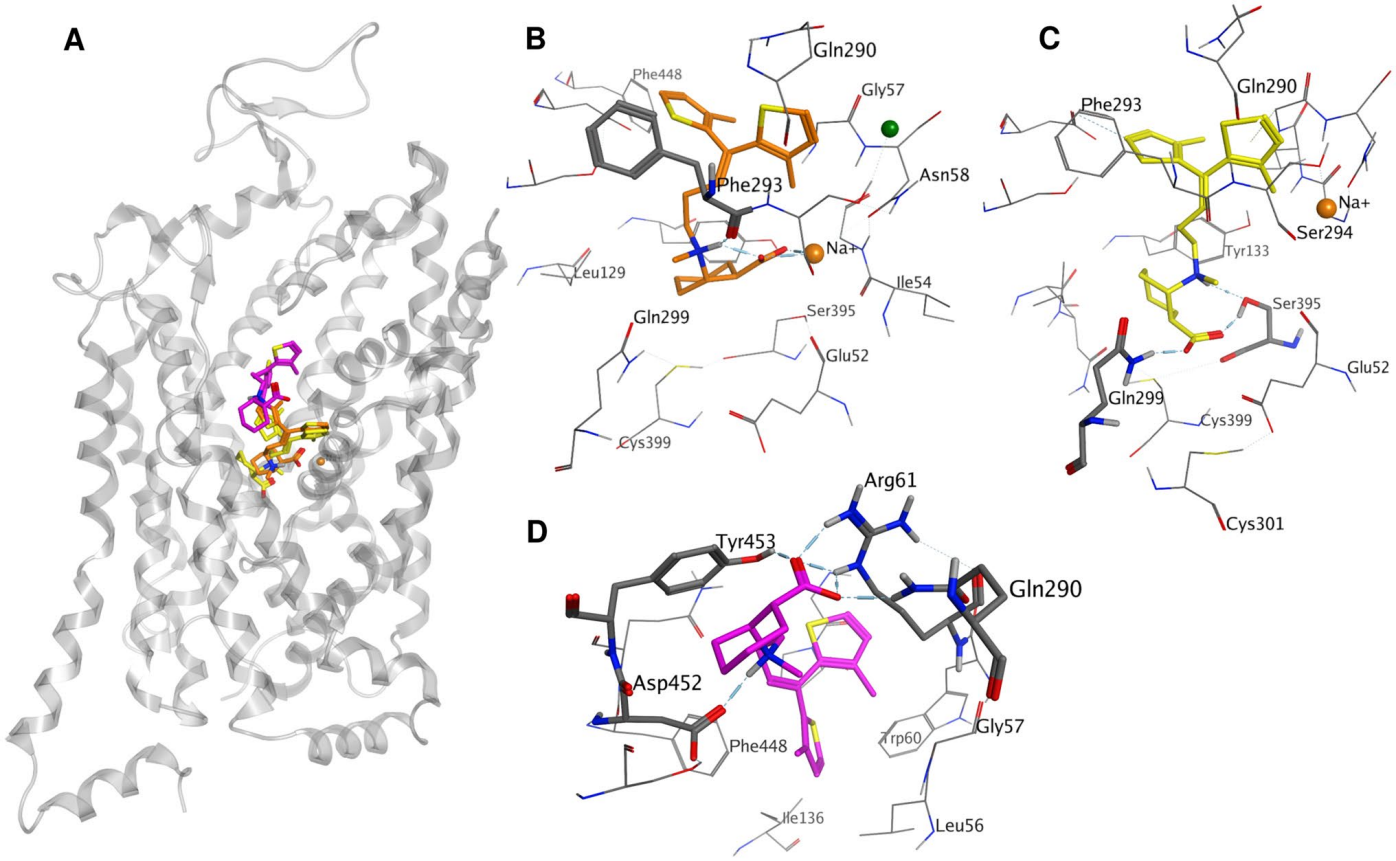
Overview of SBV2-114 docking poses in the orthosteric site (yellow and orange ligands) and allosteric site (pink ligand) in a hBGT1 homology model (**a**) [37]. **b** Docking pose of SBV2-114 according to the first binding mode in the orthosteric site. The carboxyl group coordinates a sodium ion whereas the protonated nitrogen forms a hydrogen bond with the backbone carbonyl of Phe253. The diaromatic sidechain of SBV2-114 expands into the extracellular vestibule. **c** Docking pose of the second binding mode of SBV2-114 in the orthosteric site. The carboxyl group forms a hydrogen bond with the side chain of Q299L whereas the protonated nitrogen forms a hydrogen bond with the side chain of Ser395. The diaromatic sidechain of SBV2-114 expands into the extracellular vestibule. **d** SBV2-114 docking pose in the allosteric pocket. The carboxyl group of SBV2-114 undergoes hydrogen bonding with side chains of Tyr453, Arg61 and Gln290. The protonated nitrogen forms a hydrogen bond with Asp452

To address the possibility of two binding sites for SBV2-114 at BGT1, SBV2-114 was docked into the orthosteric and allosteric site of a hBGT1 homology model based on the hSERT crystal structure PDB ID 5I73 co-crystallized with escitalopram in the orthosteric and allosteric site [15, 37]. After visually investigating the top scored poses of SBV2-114 in the ortho and allosteric site, we conclude that it is sterically possible for SBV2-114 to bind to both sites simultaneously (Fig. 3a). In more detail, two potential binding modes for SBV2-11 in the orthosteric site as well as one possible binding mode in the allosteric site was identified (Fig. 3a). The first binding mode in the orthosteric site is characterized by the carboxyl group of SBV2-114, which is a key feature of GAT inhibitors, coordinating the sodium ion (Na 1) (Fig. 3b). The positively charged nitrogen of SBV2-114, which is the second key feature of GAT inhibitors, forms a hydrogen bond with the backbone of Phe293. This residue is part of the extracellular lid which is essential for the translocation process and therefore also highly conserved among the neurotransmitter transporters [13, 48]. Finally, the lipophilic di-aromatic side chain of SBV2-114 expands into the primarily lipophilic extracellular vestibule. Since all interactions observed were either formed by functionally essential residues or ions, this binding mode was not accessible to validation by mutational experiments.

**Fig. 4:**
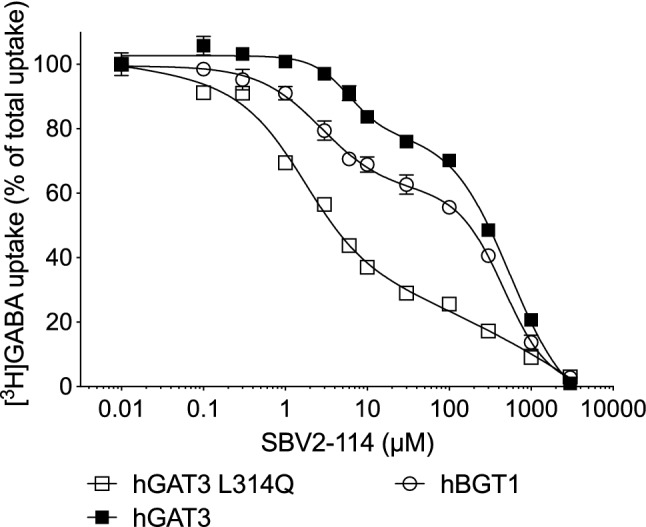
Inhibitory activity of SBV2-114 at hBGT1, hGAT3 and hGAT3 L314Q transiently expressed in tsA201 cells. SBV2-114 was tested for its ability to inhibit uptake of 30 nM [^3^H]GABA for 3 min. The experiments were performed in triplicate in two–three independent experiments and depicted as normalized means ± S.E.M (hBGT1 WT) or a representative curve with mean ± S.D. The data fitting is based on the preferred model according to the extra-sum-of-squares F test, with the biphasic fit as the preferred fit for all curves P < 0.05. See main text for IC_50_ values. Typical counts ranged from 50 to 4200 CPM

Interestingly, among the top scored poses we also observed a second binding mode in the orthosteric site where the carboxyl group of SBV2-114 forms a hydrogen bond with the side chain of glutamine 299 (Q299) and the protonated nitrogen undergoes hydrogen bonding with Ser395 (Fig. 3c). A similar alternative binding mode was already discussed by Vogensen et al. [30]. The lipophilic aromatic side chain extends analogously to the first binding mode into the extracellular vestibule. While Ser395 constitutes a highly conserved residue amongst the GATs and the monoamine transporter, Q299 is a unique residue for BGT1, corresponding to leucine in all other GATs (see alignment [46]). An interaction with this residue could possibly explain subtype selectivity and be important for the observed biphasic inhibition profile. This prompted us to investigate the activity of SBV2-114 in the hBGT1 mutant where we would predict a loss of activity. However, the [^3^H]GABA uptake results for the hBGT1 Q299L mutant showed a clear biphasic inhibition profile and no significant altered activity for SBV2-114, which would contradict the second binding mode and the relevance of Q299 for binding (data not shown). Additionally, we also investigated the activity of SBV2-114 in the corresponding hGAT3 mutant L314Q mimicking the orthosteric Q299 site of hBGT1. Through this mutation we were able to strengthen the biphasic inhibition profile of SBV2-114 and introduce activity in hGAT3 L314Q similar to hBGT1 underlining the potential relevance of Q299 for hBGT1 activity. In detail, the profile of SBV2-114 at GAT3 L314Q had a higher F value compared to at hGAT3 (15.29 vs 8.14, respectively), and hence, statistically a more pronounced biphasic CRC, and a higher Frac value compared to both hGAT3 and hBGT1 (Fig. 4).

In terms of the allosteric site (third binding mode), the best poses showed that the carboxylic group undergoes hydrogen bonding with the side chains of tyrosine 453 (Tyr453), arginine 61 (Arg61) and glutamine 290 (Gln290), whereas the protonated nitrogen forms a hydrogen bond with aspartate 452 (Asp452) (Fig. 3d). Only Tyr453 has been proposed by others to be relevant for subtype selectivity [37, 47]. Because this residue is also the only interacting residue not highly conserved among the GATs and monoamine transporters, we investigated the inhibition profile of SBV2-114 at the hBGT1 Y453A mutant. However, the [^3^H]GABA uptake results for the hBGT Y453A mutant did not show the expected decrease in activity for SBV2-114 (data not shown).

### SBV2-114 at High Concentrations Leads to an Intracellular Ca^2+^ Increase

**Fig. 5:**
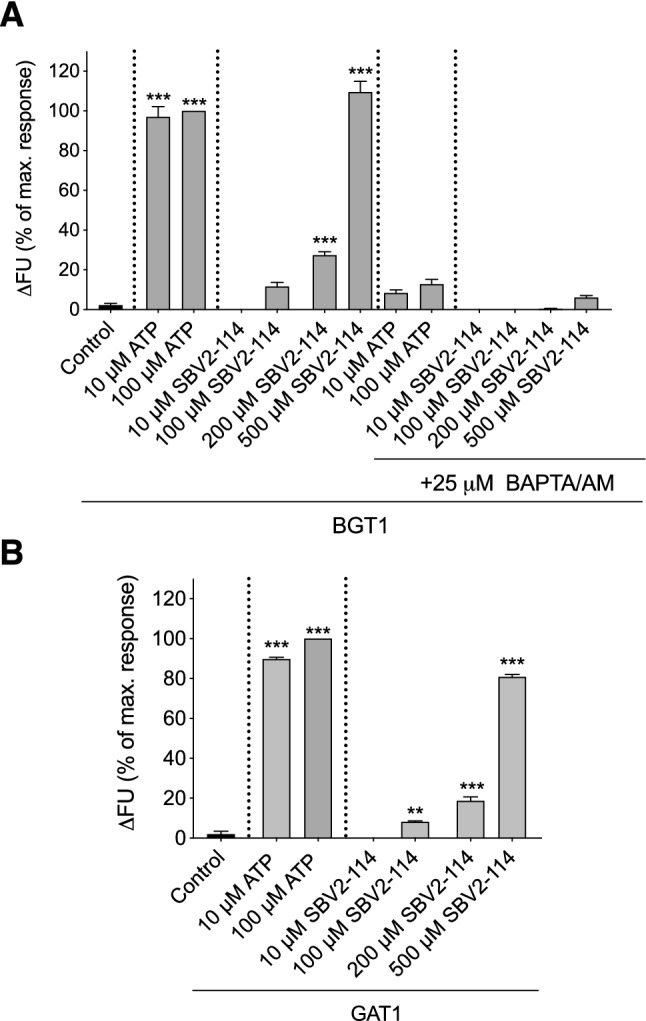
Ca^2+^ response of SBV2-114 in tsA201 cells transiently expressing hBGT1 (**a**) or hGAT1 (**b**). SBV2-114 was tested for its ability to induce an intracellular Ca^2+^ increase determined using the Fluo-4 Ca^2+^-assay with and without the presence of 25 μM BAPTA/AM. The experiments were performed in triplicate in three–four independent experiments and depicted as mean ± S.E.M. One-Way ANOVA followed by Dunnett’s multiple comparison test comparing to control levels, **P < 0.01, ***P < 0.001

A link between changes in intracellular Ca^2+^ levels and altered GAT1- and GAT3-mediated [^3^H]GABA uptake has been reported [45], which prompted us to investigate if SBV2-114 triggers an intracellular Ca^2+^ increase in tsA201 cells transiently expressing hBGT1. As depicted in Fig. 5a, SBV2-114 triggered an intracellular Ca^2+^ increase at 200 μM and 500 μM within the 3 min timeframe used for [^3^H]GABA uptake at hBGT1. As a positive control, ATP also induced a Ca^2+^ increase. Both effects could be blocked by pre-incubation with the membrane permeable Ca^2+^ chelator BAPTA/AM (Fig. 5a). A similar Ca^2+^ response was observed in tsA201 cells expressing hGAT1 (Fig. 5b), revealing that the effect was not BGT1 specific, although it is unknown if SBV2-114 triggers a Ca^2+^ response in tsA201 cells from internal stores or influx and through which target(s).

**Fig. 6:**
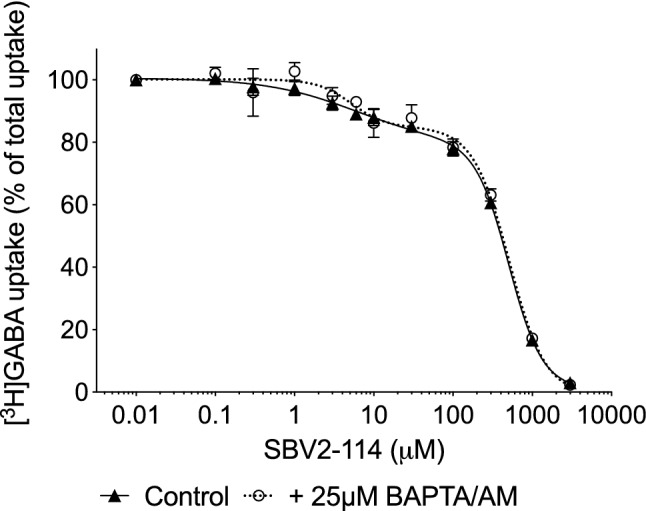
Inhibitory activity of SBV2-114 at hBGT1 transiently expressed in tsA201 cells and the effect of BAPTA/AM. Cells were pre-incubated 30 min with assay buffer ± 25 µM BAPTA/AM before SBV2-114 was tested for its ability to inhibit uptake of 30 nM [^3^H]GABA for 3 min. The experiment shown is a representative example performed in triplicate and depicted as means ± S.D. According to the extra-sum-of-squares F test, the biphasic fit was preferred for both conditions P < 0.05

Next, we wanted to investigate if the biphasic CRC of SBV2-114 was dependent on the Ca^2+^ response, and where the Ca^2+^ originates from. To this end, tsA201 cells expressing hBGT1 were pre-incubated with either the cell impermeant Ca^2+^ chelator BAPTA to abolish any possible Ca^2+^ influx or the cell permeant BAPTA/AM to chelate all intracellular Ca^2+^ increases in response to SBV2-114. It is clear from these experiments that the pre-incubation itself (30 min at 37 °C), both in the assay buffer (control) and assay buffer supplemented with BAPTA/AM, affected the inhibition curve of SBV2-114 at hBGT1 in the [^3^H]GABA uptake assay (Fig. 6), however without changing the IC_50_ values. Specifically, lowered Frac values and Hill coefficients compared to Table 1 were observed both in the control (IC_50_ = 6.1 μM and 507.0; μM, nH_1_ = -0.76, nH_2_ = -2.05; Frac = 0.21, F value = 62.75) and the BAPTA/AM experiment (IC_50_ = 5.2 μM and 512.9 μM; nH_1_ = -2.02, nH_2_ = -− 1.91; Frac = 0.15, F value = 5.61). The same scenario was observed in the BAPTA experiment (data not shown). Consequently, it is impossible to conclude on the effect of chelating Ca^2+^ in response to SBV2-114 alone. Nevertheless, although the pre-incubation affected the Frac values and Hill coefficients, the preferred fit was still biphasic in both cases (F test), altogether indicating that the Ca^2+^ response elicited by SBV2-114 is not responsible for the biphasic inhibition profile.

To address the involvement of Ca^2+^ in the biphasic profile in another way, we tested if we could abolish the biphasic profile of SBV2-114 by inhibiting any potential release of Ca^2+^ from the endoplasmic reticulum (ER), elicited by SBV2-114, by inhibiting phospholipase C (PLC), or by blocking any SBV2-114 mediated Ca^2+^ influx through the P2X7 receptor [49]. For this, tsA201 cells were pre-incubated with either U-73122 or A 804595 for 30 min prior to and during the [^3^H]GABA uptake. Inhibition of PLC with 5 μM U-73122 or P2X7 with 1 μM A 804595, however, did not convert the biphasic profile into a monophasic curve. Also, we still observed that the pre-incubation had an impact similar to pre-incubation with BAPTA/AM on the SBV2-114 inhibition curve at hBGT1 compared to Table 1 in terms of a lowered Frac value and Hill coefficients (Supplemental Fig. 1). Thus, it is not possible based on this approach either to deduce if the Ca^2+^ increase is involved in the biphasic CRC of SBV2-114 at BGT1.

In order to avoid this pre-incubation, we tested if we could induce a biphasic inhibition profile of BPDBA and GABA by triggering Ca^2+^ increases with ATP. Even though ATP modulated the BGT1 mediated [^3^H]GABA uptake and decreased the total [^3^H]GABA uptake by 9% (P < 0.001, One-way ANOVA, data not shown), the presence of ATP during the [^3^H]GABA uptake had no effect on the type of inhibition profile of BPDBA and GABA at hBGT1 (Supplemental Fig. 2).

### Anti-seizure Profile of SBV2-114

Several animal studies with the GAT1/BGT1 inhibitor EF1502 and the BGT1 inhibitor RPC-425 have reported anti-seizure effects of the compounds that cannot be ascribed solely to GAT1 [20–23]. This, together with the better selectivity of SBV2-114 (Supplemental Table 2) [46], impelled us to test the ability of SBV2-114 to prevent seizures in three mouse models. SBV2-114 was first tested in AGS Frings mice, which is a model of generalized seizures, to determine the ability of SBV2-114 to prevent sound-induced seizures and determine the approximate TPE for further testing. Frings mice were injected with 50 mg/kg SBV2-114 i.p. and tested after 15, 30, and 60 min. A subsequent time response study was conducted at a dose of 120 mg/kg and testing after 30, 60, and 120 min. The results from these studies show that SBV2-114 is brain-permeant and blocks sound-induced seizures in Frings mice in a dose-dependent manner at a TPE of 15 min (Table 3). Yet, a faster TPE cannot be excluded. In the subsequent MES test, SBV2-114 was administered at doses ranging from 50 to 200 mg/kg 15 min prior to the MES stimulus. SBV2-114 also prevented the seizures in the MES test and with an ED_50_ of 139 mg/kg. However, SBV2-114 produced rotarod impairment with a TD_50_ of 163 mg/kg, and with 95% confidence intervals overlapping with the ED_50_. In the s.c.PTZ seizure test we only examined a single higher dose of 150 mg/kg SBV2-114. At this dose, SBV2-114 did not block s.c.PTZ-induced seizures or produce rotarod impairment (Table 3). The reason for the mixed effect of SBV2-114 at rotarod impairment between the CF1 mice tested in the MES test and the s.c.PTZ seizure test is unclear. Further studies are needed to address if this could be due to e.g. sedative effects of SBV2-114.

**Table 3:** Anti-seizure effects of SBV-114 in the audiogenic induced seizure test, the MES test, and the s.c.PTZ seizure threshold test

Dose (mg/kg)	Time (min)	#Protected/#Tested	#Motor impairment/#Tested
Audiogenic induced seizure^a^			
50	15	3/4	0/3
	30	2/4	0/3
	60	1/4	0/3
	120	0/4	0/3
120	30	4/4	0/4
	60	3/4	0/4
	120	2/4	0/4
	240	1/4	0/4
Maximal electroshock seizure (MES)^b^			
50	15	0/3	0/3
100	15	0/4	0/4
125	15	2/7	0/7
150	15	4/6	2/6
200	15	4/4	3/4
s.c.PTZ seizure threshold test^c^			
150	30	0/8	0/8

Toxicity was tested in all experiments by the Rotarod test where minimal motor impairment was tested prior to the induced seizure

^a^SBV-114 (50 and 120 mg/kg) was administered i.p. and seizures were induced in AGS Frings mouse by a sound stimulus of 11 kHz (110 decibel) for 20 s at different time points. Mice were considered protected if they failed to display full hind limb tonic extension seizure

^b^SBV-114 (50, 100, 125, 150, and 200 mg/kg) was administered i.p.in CF-1 mice and seizures were induced after 15 min by electrodes placed on the eyes with a 50 mA (60 Hz) alternating current for 0.2 s. Mice were considered protected if they failed to display full hind limb tonic extension seizure

^c^SBV2-114 (150 mg/kg) was administered i.p. in CF-1 mice and seizures were induced after 15 min by a s.c. dose of PTZ (85 mg/kg). The mice were considered protected if they failed to display clonic seizures characterized by minimal forelimb clonus and/or vibrissae twitching

### Selectivity Studies of SBV2-114

Despite its dual activity at GAT1 and BGT1, EF1502 is completely devoid of any activity at a broad range of neurotransmitter systems including adrenergic, GABAergic and dopaminergic receptors in a radioligand binding study of 74 different neuroreceptors and ion channels [21]. Since SBV2-114 belongs to the same compound class as EF1502, and displayed anti-seizure effects, we wanted to address the overall selectivity of SBV2-114 in an effort to probe its potential as a tool compound to study BGT1 function in vivo. Hence, the selectivity of SBV2-114 for BGT1 over 42 neuroreceptors and transporters was tested in a similar manner as EF1502 through an affinity screening program supported by NIMH PDSP. From this evaluation, it is clear that SBV2-114 is fairly nonselective and competes with the radioligand binding to a number of other known drug targets including the serotonin receptors (5-HT_2B_ 5-HT_2C_, 5-HT_5A_, and 5-HT_6_), the adrenergic receptor alpha_2A_, and the dopamine transporter (DAT) (see Supplemental Table 4 for details) consequently hampering the usability of SBV2-114 to study BGT1 function in vivo. Additional studies are needed to investigate if interaction with any of these targets are implicated in the biphasic inhibition profile of SBV2-114 at hBGT1 in which case they would have to be endogenously expressed in our cell lines. Most likely, the involvement of any serotonin receptors and the adrenergic receptor alpha_2A_ can be disregarded, since they are not endogenously expressed in HEK-293 cells [50].

## Discussion

Based on the detailed molecular characterization of SBV2-114 using the [^3^H]GABA uptake assay, it is clear that SBV2-114 inhibits the GATs in an atypical manner. We have demonstrated an apparently novel biphasic inhibition profile of SBV2-114, which is most evident at BGT1. At BGT1, SBV2-114 has an IC_50_ value in the low μM range (m/hBGT1: 3.9/4.7 μM) inhibiting a fraction of 0.42–0.45 of the BGT1-mediated GABA uptake, while the second IC_50_ is in the high μM range (m/hBGT1: 402.7/555.9 μM). Additionally, we show that the high affinity component of the biphasic profile of SBV2-114 was most evident when the Frac value (% inhibition) and potency was high, as is the case for BGT1 compared to the other GATs. We further show that (*R*)-EF1502 and other SBV2-114 analogues, all sharing the β-amino acid moiety attached to di-aromatic lipophilic side-chains, inhibit BGT1 in a biphasic manner. The reason why there are no prior notions of (*R*)-EF1502 having a biphasic profile is likely due to the fact that the [^3^H]GABA uptake assay is typically performed with 8-point CRCs and not 12 and is thus problematic to capture. By contrast, BGT1 inhibitors such as BPDBA and NNC 05-2090, which comprise different scaffolds compared to SBV2-114, as well as GABA itself, display regular monophasic inhibition curves, supporting the finding that the biphasic profile at BGT1 is reserved to the class of structurally related compounds to SBV2-114 only. Importantly, we show that this biphasic inhibition profile of SBV2-114 at BGT1 in [^3^H]GABA uptake is indeed a true phenomenon as it occurs regardless of species, origin of the transporters (recombinant vs endogenous), expression levels, and kinetics.

Recently, it was demonstrated that the highly related *human* serotonin transporter comprises an additional allosteric binding site in the extracellular vestibule above the orthosteric site [15]. This prompted us to investigate whether a similar allosteric site exists in BGT1 which could accommodate SBV2-114, and hence, could explain the observed biphasic behaviour. With the help of a homology model based on the SERT crystal structure along with docking studies, we identified hBGT1 Q299L in the orthosteric site and hBGT1 Tyr453 in the allosteric site to be possibly involved in SBV2-114 binding (Fig. 3). Furthermore, the top scored docking poses of SBV2-114 in the orthosteric and allosteric site suggested that it is sterically feasible for SBV2-114 to bind to both sites simultaneously (Fig. 3a). Nevertheless, we were not able to alter the biphasic inhibition profile of SBV2-114 by introducing a single mutation in the orthosteric site (hBGT1 Q299L) or the allosteric site (hBGT1 Y453A). However, by introducing a glutamine in hGAT3 at the position L314Q, which corresponds to Q299L in the orthosteric site of hBGT1, an increase in the activity of SBV2-114 and a strengthening of the biphasic profile was observed. Thus, residue Q299 in hBGT1 and residue L314 in hGAT3 appear to be important for the binding and activity of SBV2-114, but not solely responsible for the biphasic inhibition profile considering the unaltered inhibition profile of SBV2-114 at hBGT1 Q299L. The latter could be the result of possible compensation since the docking studies suggest that SBV2-114 can adopt two slightly different binding modes in the orthosteric pocket where the carboxyl group of SBV2-114 either interacts with the sodium ion Na1 or undergoes hydrogen bonding with Q299L (Fig. 3b and c). Lastly, the [^3^H]GABA uptake data clearly shows that SBV2-114 inhibits GATs in an atypical manner, and hence, it is possible that other interacting residues not specific to BGT1 are accountable for the biphasic inhibition profile of SBV2-114.

To address the link between Ca^2+^, altered [^3^H]GABA uptake [45], and the potential involvement in the biphasic inhibition profile of SBV2-114, we first demonstrated that SBV2-114 triggered an intracellular Ca^2+^ increase at high concentrations independent of BGT1, via an unknown mechanism. Additionally, we showed that ATP, which triggered a large increase in intracellular Ca^2+^ levels, lowered the BGT1-mediated [^3^H]GABA uptake, supporting that a change in intracellular Ca^2+^ levels modulate BGT1 activity as reported for GAT1 and GAT3 [45]. Nevertheless, the subsequent studies aimed at reducing the Ca^2+^ involvement in the biphasic inhibition profile of SBV2-114 at hBGT1 were inconclusive. Thus, further studies are needed to investigate if the intracellular Ca^2+^ increase triggered by the high concentrations of SBV2-114 is implicated in the observed biphasic behaviour, especially since the least potent part of the biphasic inhibition curve lies in the same range as the Ca^2+^ releasing concentrations of SBV2-114.

Although a biphasic inhibition profile for the subfamily of GABA transporters has not been noted before, the phenomenon has been described within the family of SLC6A transporters [51–54]. A biphasic activity of a small molecule ATM7 has been reported at SERT, and was explained by an enhanced SERT uptake in response to nM concentrations of ATM7, while ATM7 inhibited SERT uptake at μM concentrations [53], with no notion of two binding sites for ATM7 at SERT. A mechanism like that for SBV2-114, however, is not in agreement with the [^3^H]GABA uptake data presented here, since nM concentrations of SBV2-114 decreased the total [^3^H]GABA uptake. Another example comes from a truncated version of the *human* DAT, which displayed a biphasic inhibition profile of [^3^H]DA uptake presumably due to two DAT populations with different affinities for dopamine [51, 55]. The fact that a biphasic inhibition profile of SBV2-114 was observed in cells transiently expressing BGT1 argues against the possibility that two BGT1 populations underlie the biphasic profile observed. Last, it seems unlikely that the presumed dimerization of BGT1 [56, 57] accounts for the biphasic profile of SBV2-114 at BGT1, since other BGT1 inhibitors including NNC 05-2090, BPDBA as well as GABA inhibit BGT1 in a monophasic manner. If not, then a specific binding mode of SBV2-114 to the dimer should be in play.

BGT1 has attracted attention as a potential target for seizure management due to animal studies demonstrating anti-seizure properties of the GAT1/BGT1 inhibitor EF1502 and the BGT1 inhibitor RPC-425 [20–23]. Still, the unaltered seizure susceptibility reported in BGT1 KO mice has questioned the importance of BGT1 as such [26], and BGT1 selective compounds are warranted to clarify the role of BGT1 in epilepsy. As reported here, SBV2-114 is indeed more selective for mBGT1 over mGAT1-3 (IC_50_ > 1000 μM) as compared to both EF1502 and RPC-425 [46], which is an intriguing finding demonstrating how much a small chemical change from EF1502 to SBV2-114 can affect selectivity. The improved BGT1 preference of SBV2-114 encouraged us to test the anti-seizure properties of SBV2-114. We report that SBV2-114 dose-dependently prevented both audiogenic induced seizures in Frings mice and maximal electroshock induced seizures in CF-1 mice. In contrast, SBV2-114 did not prevent a minimal clonic seizure in the s.c.PTZ test at the dose (150 mg/kg, i.p) and pre-treatment time (30 min) tested. Though, this might be due to the apparent fast pharmacokinetics of SBV2-114 according to the testing in Frings mice illustrating that SBV2-114 was most effective at the earliest time point tested (15 min). Despite SBV2-114’s preference for mBGT1 over mGAT1-3, results from the NIMH PDSP screen of neuro targets suggest that SBV2-114 also interacts with other molecular targets; a finding that renders it difficult to conclude that the anti-seizure effect of SBV2-114 is solely related to BGT1 inhibition. Nonetheless, the only known shared target for EF1502, RPC-425, and SBV2-114 is BGT. This, coupled with the fact that EF1502 has a good overall selectivity profile [21], collectively supports a role for BGT1 in epilepsy. This is in agreement with the synergistic effects of EF1502 and tiagabine in epilepsy models that cannot be linked to GAT1 exclusively [20–23]. Of note, the effect of combining RPC-425 and tiagabine did not have synergistic but only additive effects, which might be explained by differences in the pharmacokinetics between EF1502 and RPC-425 [20] rather than dismissing the importance of BGT1 in epilepsy.

## Future Directions

While the molecular mechanism underlying the biphasic inhibition profile of SBV2-114 at BGT1 remains elusive, we show that it is a consistent phenomenon for this class of compounds all sharing a β-amino acid moiety attached to di-aromatic lipophilic side-chains. Although the in vivo suitability of SBV2-114 is poor due to its low selectivity and narrow therapeutic index, the data support previous findings suggesting an anti-seizure role for BGT1 inhibition. Further, SBV2-114 still constitutes a valuable in vitro tool compound to identify molecular mechanisms modulating GABA uptake, specifically the biphasic inhibition profile, and as a lead compound for the design of more selective BGT1 inhibitors. Ideally such studies would be aided by a BGT1 inhibitor radioligand for binding studies.

## Electronic supplementary material

Below is the link to the electronic supplementary material.

Supplementary file1 Supplemental Fig. 1 Inhibitory activity of SBV2-114 at BGT1 transiently expressed in tsA201 cells in the presence of 5 μM U-73122 or 1 μM A 804598. Cells were pre-incubated 30 min with U-73122 (a) or A 804598 (b) before SBV2-114 was tested for its ability to inhibit uptake of 30 nM [3H]GABA for 3 min in the presence of U-73122 and A 804598. All experiments were performed in triplicate in two-four independent experiments. The curve fittings preferred according to the extra-sum-of-squares F test were in all cases the biphasic fit, P<0.001. (a) Control: F value=8.77, U-73122: F-value=9.35; (b) Control: F value=34.71, A 804598: F-value=10.48 (EPS 239 kb)

Supplementary file2 Supplemental Fig. 2 Inhibitory activity of GABA and BPDBA at hBGT1 transiently expressed in tsA201 cells in the presence of 10 μM ATP. GABA (a) and BPDBA (b) were tested for their ability to inhibit uptake of 30 nM [3H]GABA for 3 min. All experiments were performed in triplicate in two-three independent experiments. The curve fittings preferred according to the extra-sum-of-squares F test were in all cases the monophasic fit, P>0.05 (EPS 268 kb)

Supplementary file3 (DOCX 17 kb)
