# Supplementary material for: Pharmacological Characterization of a Betaine/GABA Transporter 1 (BGT1) Inhibitor Displaying an Unusual Biphasic Inhibition Profile and Anti-seizure Effects
Source: Neurochem Res. 2020 Apr 4;45(7):1551–65. doi: 10.1007/s11064-020-03017-y (PMC7297817; doi:10.1007/s11064-020-03017-y)
Supplement: Supplementary file 3 — Supplementary file3 (DOCX 17 kb) [file 11064_2020_3017_MOESM3_ESM.docx]

**Supplemental Table 1** Inhibitory activity of SBV2-114 at hBGT1 transiently expressed in tsA201 cells using varying amounts of plasmid DNA. SBV2-114 was tested for its ability to inhibit uptake of 30 nM [^3^H]GABA for 3 min. All experiments were performed in triplicate in two-five independent experiments. The data fitting is based on the preferred model according to the extra-sum-of-squares F test, and the associated P, F, DFn,DFd, and Frac values are indicated.

| [^3^H] GABA uptake at hBGT1 | | | Extra-sum-of-squares F test | | | | | |
| --- | --- | --- | --- | --- | --- | --- | --- | --- |
| cDNA (μg) | IC_50_ (pIC_50_ ± S.E.M.) (μM) | | P value | F value | DFn,DFd | Frac | nH_1_, nH_2_ |  |
| **2** (n=2) | 3.4  (5.47 ± 0.23) | 517.6  (3.29 ± 0.03) | 0.002 | 8.01 | 3, 17 | 0.44 | -0.86,-1.75 |  |
| **4** (n=2) | 2.2  (5.66 ± 0.20) | 514.0  (3.29 ± 0.04) | <0.001 | 16.05 | 3, 17 | 0.32 | -1.31,1.62 |  |
| **8** (n=5) | 4.7  (5.28 ± 0.10) | 555.9  (3.26 ± 0.03) | <0.001 | 11.19 | 3, 53 | 0.42 | -0.86,-2.13 |  |

**Supplemental Table 2** Functional characterization of SBV2-114 at mGATs stably expressed in HEK-293 cells. SBV2-114 was tested for its ability to inhibit uptake of 30 nM [^3^H]GABA for 3 min. and analysis of the preferred curve fitting model is indicated. The data fitting is based on the preferred model according to the extra-sum-of-squares F test, and the number of experiments that preferred the given fit is indicated (N) as well as the associated Frac values when applicable.

| [^3^H] GABA uptake at mGATs stably expressed in HEK-293 cells | | | | | |  |
| --- | --- | --- | --- | --- | --- | --- |
| Sybtype | IC_50_ (pIC_50_ ± S.E.M.) (μM) | | N | Frac | Fit | |
| mBGT1 | 3.8  (5.42 ± 0.08) | 408.3  (3.39 ± 0.03 | 5 | 10.6 | Biphasic | |
| mGAT1 | 0.42  (6.38) | 755.09  (3.12) | 1 | 0.14 | Biphasic | |
|  | >1,000 | | 1 | n.a. | Monophasic | |
|  | >1,000 | | 1 | n.a. | Ambiguous | |
| mGAT2 | >1,000 | | 3 | n.a. | Ambiguous | |
| mGAT3 | >1,000 | | 3 | n.a. | Ambiguous | |

**Supplemental Table 3** Functional characterization of SBV2-114 at hGATs stably expressed in CHO cells. SBV2-114 was tested for its ability to inhibit uptake of 30 nM [^3^H]GABA for 3 min. and analysis of the preferred curve fitting model is indicated. The data fitting is based on the preferred model according to the extra-sum-of-squares F test, and the number of experiments that preferred the given fit is indicated (N) as well as the associated Frac values when applicable.

| [^3^H] GABA uptake at hGATs stably expressed in CHO cells | | | | |  |
| --- | --- | --- | --- | --- | --- |
| Sybtype | IC_50_ (pIC_50_ ± S.E.M.) (μM) | | N | Frac | Fit |
| hBGT1 | 0.05  (7.29 ± 0.75) | 489.78  (3.31 ± 0.00) | 2 | 0.10 | Biphasic |
| hBGT1 | 446.68  (3.35 ± 0.02) | | 2 | n.a. |  |
| hGAT1 | 565.80  (3.25 ± 0.02) | | 2 | n.a. | Ambiguous |
|  | 476.2  (3.32) | | 1 | n.a. | Monophasic |
| hGAT2 | 3.31  (5.48) | 549.54  (3.26) | 1 | 0.06 | Biphasic |
| hGAT2 | 524.81  (3.28 ± 0.02) | | 3 | n.a. | Ambiguous |
| hGAT3 | 2.09  (5.68 ± 0.31) | 602.56  (3.22 ± 0.31) | 3 | 0.09 | Biphasic |

**Supplemental Table 4** Selectivity of SBV2-114 for BGT1 over 42 different neuroreceptors and transporters. SBV2-114 was tested in radioligand binding assays at a concentration of 10 μM followed up by full curves if inhibition exceeded 50% at 10 μM, n=4. IC_50_ values in μM (K_i_ μM)^a^. The targets in bold were inhibited by >35% at 10 μM.

| % inhibition (K_i_ μM) in radioligand binding assays | | |  |
| --- | --- | --- | --- |
| Target | Radioligand | SBV2-114 | |
| 5-HT1A | [^3^H]8-OH-PDAT | -7.9 | |
| 5-HT1B | [^3^H]GR125743 |  | |
| 5-HT1D | [^3^H]GR125743 | 19.6 | |
| 5-HT1E | [^3^H]5-HT | -12.9 | |
| 5-HT2A | [^3^H]ketanserin | 20.0 | |
| 5-HT2B | [^3^H]LSD | **96.8 (0.42)** | |
| 5-HT2C | [^3^H]mesulergine | **77.5 (0.67)** | |
| 5-HT3 | [^3^H]LY278584 | -3.0 | |
| 5-HT5A | [^3^H]LSD | **36.6** | |
| 5-HT6 | [^3^H]LSD | **50.0 (10.0 ave)** | |
| 5-HT7 | [^3^H]LSD | 14.1 | |
| D1 | [^3^H]SCH23390 | 14.0 | |
| D2 | [^3^H]N-methylspiperone | 12.3 | |
| D3 | [^3^H]N-methylspiperone | 27.8 | |
| D4 | [^3^H]N-methylspiperone | -5.1 | |
| D5 | [^3^H]SCH23390 | **51.7 (10.0 ave)** | |
| Sigma1 | [^3^H]pentazocine(+) | **55.6 (0.41)** | |
| Sigma2 | [^3^H]DTG | **48.3** | |
| Alpha1A | [^3^H]prazosine | 0.1 | |
| Alpha1B | [^3^H]prazosine | 1.9 | |
| Alpha1D | [^3^H]prazosine | -4.7 | |
| Alpha2A | [^3^H]rauwolscine | **42.8** | |
| Alpha2B | [^3^H]rauwolscine | 24.9 | |
| Alpha2C | [^3^H]rauwolscine |  | |
| Beta1 | [^125^I]pindolol | **37.9** | |
| Beta2 | [^3^H]CGP12177 |  | |
| Beta3 | [^3^H]CGP12177 | 8.3 | |
| BZP rat brain | [^3^H]Flunitrazepam | 9.1 | |
| GABA_A_ rat brain | [^3^H]muscimol | -1.5 | |
| DAT | [^3^H]WIN35428 | **36.9** | |
| NET | [^3^H]nisoxetine | 0.1 | |
| SERT | [^3^H]citalopram | 18.8 | |
| DOR | [^3^H]DADLE | **36.6** | |
| H1 | [^3^H]pyrilamine | **51.2** | |
| M1 | [^3^H]QNB | 18.3 | |
| M2 | [^3^H]QNB | -1.9 | |
| M3 | [^3^H]QNB | **55.4** | |
| M4 | [^3^H]QNB | -5.7 | |
| M5 | [^3^H]QNB | 23.5 | |
| KOR | [^3^H]U69593 (2007-07-27) | **40.8** | |
| MOR | [^3^H]DAMGO (2007-07-27) | **42.0** | |

^a^K_i_ determinations, agonist and/or antagonist functional data and receptor binding profiles were generously provided by the National Institute of Mental Health’s Psychoactive Drug Screening Program (NIMH PDSP), Contract # HHSN-271-2008-00025-C).
